# Flazin as a Lipid Droplet Regulator against Lipid Disorders

**DOI:** 10.3390/nu14071501

**Published:** 2022-04-03

**Authors:** Xunzhi Wu, Zhen Chen, Yue Wu, Yifan Chen, Jiaping Jia, Nianqiu Shen, Hitoshi Chiba, Shu-Ping Hui

**Affiliations:** 1 Faculty of Health Sciences, Hokkaido University, Kita-12, Nishi-5, Kita-ku, Sapporo 060-0812, Japan; xunzhi.wu.m6@elms.hokudai.ac.jp (X.W.); chenzhen@hs.hokudai.ac.jp (Z.C.); wuyue123@hs.hokudai.ac.jp (Y.W.); ovd61363@elms.hokudai.ac.jp (Y.C.); jiaping.jia.s1@elms.hokudai.ac.jp (J.J.); nianqiu.shen.z0@elms.hokudai.ac.jp (N.S.); 2 Department of Nutrition, Sapporo University of Health Sciences, Nakanuma Nishi-4-2-1-15, Higashi-ku, Sapporo 007-0894, Japan; chiba-h@sapporo-hokeniryou-u.ac.jp

**Keywords:** lipid-storage disorders, metabolic diseases, diabetic nephropathy, nutraceuticals, functional foods, triglyceride, lipid metabolism, lipidomics, mass spectrometry

## Abstract

Lipid disorders are closely related to numerous metabolic diseases, and lipid droplets (LDs) have been considered as a new target for regulating lipid metabolism. Dietary intervention and nutraceuticals provide safe and long-term beneficial effects for treating metabolic diseases. Flazin is a diet-derived bioactive constituent mainly existing in fermented foods, of which the lipid metabolism improvement function has not been studied. In this study, the effect of flazin on lipid regulation at both cell level and organelle level was investigated. Lipidomic profiling showed that flazin significantly decreased cellular triglyceride (TG) by 12.0–22.4% compared with modeling groups and improved the TG and free fatty acid profile. LD staining revealed that flazin efficiently reduced both cellular neutral lipid content by 17.4–53.9% and LD size by 10.0–35.3%. Furthermore, nanoelectrospray ionization mass spectrometry analysis proved that flazin exhibited a preferential suppression of LD TG and regulated LD morphology, including a size decrease and surface property improvement. An evaluation of related gene expression suggested the mechanism to be lipolysis promotion and lipogenesis inhibition. These findings indicated that flazin might be an LD regulator for reversing lipid metabolism disturbance. Moreover, the strategy proposed in this study may contribute to developing other nutraceuticals for treating lipid disorder-related metabolic diseases.

## 1. Introduction

Dysregulation of lipid metabolism has been verified as the main cause of many metabolic diseases, including diabetic nephropathy (DN), atherosclerosis, non-alcoholic fatty liver disease, and others [1,2,3,4]. The prevalence of these metabolic diseases is increasing along with the global pandemic of obesity [5]. In a state of obesity, the energy intake exceeds the storage capacity of adipose tissue, leading to the accumulation of the overloaded energy as fat packed in lipid droplets (LDs) to non-adipose tissue, which is also known as ectopic lipid accumulation (ELA) [6].

LDs not only serve as a reservoir for lipid deposition but act a crucial role in maintaining lipid homeostasis through lipid synthesis, metabolism, and transportation. It was reported that, during DN, LDs accumulate in the tubular portion of the kidney, which is one of the main sites of DN lesions [7]. This lipid-storage disorder induces lipotoxicity by promoting inflammation, producing reactive oxygen species, and causing cell death [8]. The increasing knowledge on the association between ELA and DN has driven researchers to consider novel therapeutic strategies for DN that aim to regulate LDs, reverse lipid disorder, and maintain lipid homeostasis.

**Figure 1:**
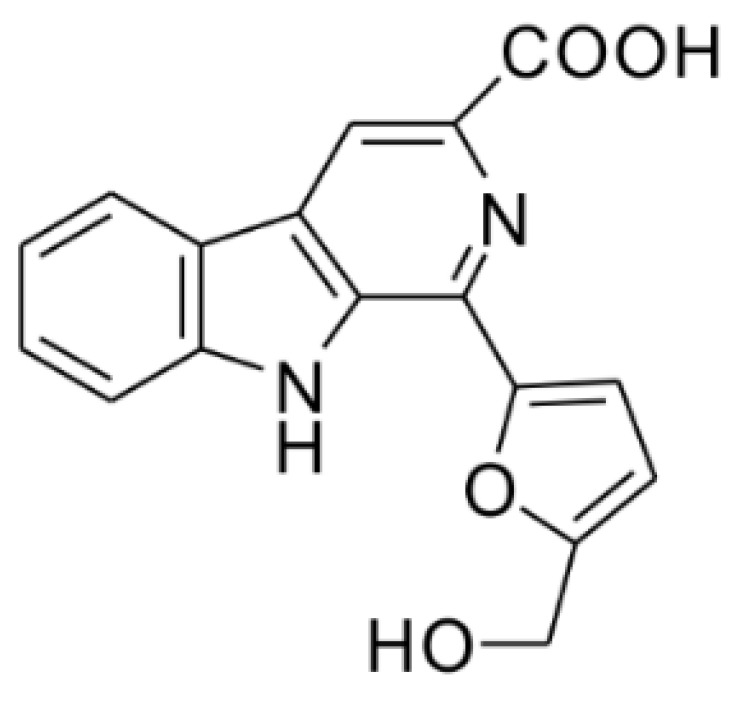
Chemical structure of flazin.

An appropriate diet is one of the major determinants of maintaining optimal health [2]. Diet-derived bioactive components also receive a long-lasting focus on their nutraceutical potential in treating metabolic diseases [9,10]. Flazin, as a diet-derived β-carboline alkaloid, is mainly found in fermented foods (e.g., sake, rice vinegar, and soy sauce) [11] and fruit juice (e.g., cherry tomato, black currant, and Nitraria tangutorum) [12,13,14], and its structure is shown in Figure 1. In terms of the health-beneficial functions of flazin, there have been studies that claim its anti-HIV [15], antioxidant [16], and immunomodulatory [17] activities. However, to the best of our knowledge, flazin has not yet been studied for its effect on lipid metabolism regulation.

This study aimed to investigate the effect of flazin on the lipid metabolism of cells under the overloading of fats. Moreover, considering the crucial role of LDs in lipid metabolism, the present study further focused on the dynamics of LDs of cells with flazin treatment. Palmitic acid (PA) and oleic acid (OA), two of the most abundant fatty acids in the diet [18], were selected to induce lipid overloading in the human kidney proximal tubular epithelial (HK-2) cell line. At the same time, flazin was co-treated to attenuate the lipid accumulation-induced disorders. The cellular lipid profile, the LD lipid profile, and LD morphology were determined using a combined approach of liquid chromatography-tandem mass spectrometry (LC-MS/MS), nanoelectrospray ionization mass spectrometry (nanoESI-MS), and organelle staining. Furthermore, as the underlying mechanism, the expression changes of lipogenic and lipolytic genes were also assessed and discussed.

## 2. Materials and Methods

### 2.1. Chemicals and Reagents

For flazin synthesis, L-tryptophan and 5-(hydroxymethyl)furfural were purchased from Sigma-Aldrich (Saint Louis, MO, USA). For cell culture and biological assays, phosphate-buffered saline (PBS), Dulbecco’s modified Eagle’s medium (DMEM), and fatty acid-free bovine serum albumin (BSA) were purchased from Nacalai Tesque (Kyoto, Japan); fetal bovine serum (FBS) was purchased from Gibco (Grand Island, NY, USA); penicillin-streptomycin (100 U/mL) was purchased from Fujifilm Wako (Osaka, Japan). For MS analysis, free fatty acid (FFA) 18:1-d9, triglyceride (TG) 15:0/18:1-d7/15:0, and TG 11:0/11:0/11:0 were obtained from Sigma-Aldrich. Unless otherwise stated, other chemicals were purchased from Kanto Chemical (Tokyo, Japan).

Flazin was prepared by chemical synthesis according to the previously reported method [15] with a slight modification. The detailed synthetic route is described in the Supplementary Material and shown in Figure S1. To validate the structure and purity, we performed nuclear magnetic resonance (NMR) using a JNM-ECP 400 NMR spectrometer (400 MHz, JEOL, Tokyo, Japan) and the mass spectrometry (MS) tests using a Thermo LXQ linear ion-trap mass spectrometer (Thermo Fisher Scientific Inc., San Jose, CA, USA), respectively. The following data were obtained: ^1^H NMR (DMSO-d6, 400 MHz): δ 11.58 (1H, s), 8.84 (1H, s), 8.42 (1H, d, *J* = 8.3 Hz), 7.82 (1H, d, *J* = 8.8 Hz), 7.65 (1H, t, *J* = 7.3 Hz), 7.43 (1H, d, *J* = 3.2 Hz), 7.35 (1H, t, *J* = 8.0 Hz), 6.63 (1H, d, *J* = 3.4 Hz), 4.69 (2H, s) (Figure S2A); ^13^C NMR (DMSO-d6, 100 MHz): δ 167.2, 157.8, 151.8, 142.0, 138.0, 133.0, 132.4, 130.4, 129.4, 122.5, 121.5, 121.1, 116.2, 113.4, 111.6, 109.8, 56.5 (Figure S2B); positive ESI-MS signals: *m/z*: 309.1 (100, [M + H]^+^), 331.1 (15, [M + Na]^+^) (Figure S3A); negative ESI-MS signals: *m/z* 307.1 (100, [M − H]^−^), 343.1 (65, [M + Cl]^−^) (Figure S3B). These data agreed with the literature [19,20], indicating the identity and authenticity of the prepared flazin. The purity of prepared flazin was estimated to be higher than 95% based on MS and NMR data.

### 2.2. Cell Culture and Treatment

The HK-2 cell line, obtained from American Type Culture Collection (Manassas, VA, USA), was cultured in DMEM (10% fetal bovine serum and 1% penicillin/streptomycin) with 5% CO_2_ at 37 °C in a humidified incubator. PA and OA were firstly conjugated to fatty acid-free BSA following the previously described method with some modifications [21]. Briefly, 500 mmol of fatty acid was added into 1 mL of ethanol at 37 °C and mixed until completely dissolved. The fatty acid solution was transferred to DMEM containing 10% fatty acid-free BSA, and the final molar ratio of fatty acid to BSA was 3.3:1.

For testing cell viability, cells were seeded in a 96-well plate with 1 × 10^4^ cells per well and cultured overnight. Then, different concentrations of PA, OA, or flazin were introduced and incubated for 24 h. According to the manufacturer’s instruction, cell viability was assessed with a cell counting kit-8 (CCK-8) (Dojindo, Kumamoto, Japan).

For the other biological experiments, the cells were divided into different groups as follows: (1) control group: cells were incubated with 0.4% BSA; (2) modeling groups: cells were incubated with PA or OA (200 μM) for 24 h, defined as PA group or OA group; (3) treatment groups: cells were pretreated with different concentration of flazin (40 μM or 80 μM) and then incubated with PA or OA (200 μM), described as PA-F40, PA-F80, OA-F40, or OA-F80 groups. For testing bioactivities, flazin was first dissolved in DMSO and then added to the culture medium, making a final DMSO concentration of 0.05%.

### 2.3. Cellular Lipid Extraction

Cells were seeded with 1 × 10^5^ cells per dish. After different treatments, the culture medium was removed, and cells were washed with PBS. Cells were collected by a scraper, and the total lipid was extracted according to the previously reported method [22] with some modifications. Briefly, the cells from each dish were transferred to a 1.5 mL Eppendorf^®^ tube. Then, the cells were extracted with 900 μL of MTBE/MeOH/H_2_O 6:2:1 (v/v, with 400 pmol of TG 15:0/18:1-d7/15:0 and 600 pmol of FFA 18:1-d9 as internal standard (IS)) twice, followed by centrifugation, collection of the organic phase, and drying under vacuum. The obtained cellular lipids were dissolved in MeOH and stored at −80 °C until analysis.

### 2.4. LC/MS and MS/MS Analysis

The semi-quantitative analysis of the cellular lipids was performed using a Prominence HPLC system (Shimadzu Corp., Kyoto, Japan) coupled to an LTQ Orbitrap mass spectrometer. The conditions were according to our previous work [23]. In brief, an Atlantis T3 C18 column (2.1 mm × 150 mm, 3 µm; Waters, Milford, MA, USA) was employed for chromatographic separation, and the electrospray ionization (ESI) source in both positive and negative modes was equipped for detection. The MS/MS fragmentation was performed using collision-induced dissociation (CID) by data-dependent acquisition. For raw data processing, Xcalibur 2.3 (Thermo Fisher Scientific) was used with the help of the LIPIDMAPS database (www.lipidmaps.org, accessed on 12 February 2022) and our in-house library [23]. The lipid data were normalized by total protein level, which was determined using the Pierce BCA Protein Assay Kit (Thermo Fisher Scientific), and the final results are presented as relative amounts to the control group. The amount of each lipid species was calculated with the equation below:

$${\mathrm{Amount}}_{\mathrm{analyte}}={\mathrm{Amount}}_{\mathrm{IS}}\;\times \;{\mathrm{Peak}\;\mathrm{area}}_{\mathrm{analyte}}/{\mathrm{Peak}\;\mathrm{area}}_{\mathrm{IS}}$$

Additionally, the total TG and total FFA were calculated as the sum of all the TG and FFA species, respectively, listed in Table S1.

### 2.5. Oil Red O Staining

Oil red O is a fat-soluble dye used extensively for neutral lipid staining, of which the stock solution was prepared by adding 150 mg of oil red O powder to 50 mL of isopropanol. The oil red O working solution was freshly prepared by mixing 15 mL of oil red O stock solution with 10 mL of deionized water. The solution was left undisturbed for 10 min and then filtered with a Millipore Millex-HV 0.45-μm filter (Bedford, MA, USA). Before staining, the culture medium was removed, and cells were rinsed with PBS. For fixing the cells, 10% formalin was added, and cells were incubated for 30 min. Then, cells were washed with PBS and immersed in oil red O working solution for 20 min. The cells were subsequently washed with water to remove excess stain and observed under an IX71 microscope (Olympus, Tokyo, Japan). The lipid contents and LD size in HK-2 cells were determined using ImageJ software [24].

### 2.6. LD Analysis by NanoESI-MS

LD sampling was performed according to the previously reported method [25] with slight modification. For semi-quantification of TG in LDs, nanotips were preloaded with 40 pmol of TG 11:0/11:0/11:0 (in 5 μL MeOH) as the IS, and the solvent was volatilized in nitrogen. Before LD extraction, the culture medium was discarded and replaced with 160 mM of NH_4_COOH solution. The operation was aided by an IM-11 three-dimensional mobile manipulator (Narishige, Tokyo, Japan) and guided by an inverted IX71 microscope in a bright field. LDs were aspirated into nanotips, and the nanotips were then backfilled with 5 μL of organic solvent (MeOH/iPrOH = 1:9 v/v, with 0.1% trifluoroacetate). The detection was conducted on an LTQ Orbitrap mass spectrometer with a nanoESI source, of which the instrument parameters were the same as described previously [25]. For semi-quantitative comparison, the total levels of TG, phosphatidylcholine (PC), and phosphatidylethanolamine (PE) were calculated as the sum of all the molecular species within the same class.

### 2.7. Reverse Transcription-Quantitative Polymerase Chain Reaction (RT-qPCR)

Gene expressions of glyceraldehyde-3-phosphate dehydrogenase (GAPDH), adipose triglyceride lipase (ATGL), acetyl-CoA carboxylase (ACC), fatty acid synthase (FAS), and stearoyl-CoA desaturase-1 (SCD-1) were tested using RT-qPCR. According to the method described in the manufacturer’s instructions, total RNA was isolated from cells by TRIzol method (Invitrogen, Waltham, MA, USA). Afterward, RT-PCR was performed using ReverTra Ace (Toyobo Co., Ltd., Osaka, Japan) following the manufacturer’s instructions. QPCR was then carried out using Fast SYBR Green Master Mix (Applied Biosystems, Waltham, MA, USA) in a Step One Plus Real-Time PCR system (Applied Biosystems) to determine mRNA expressions of target genes. The thermocycling conditions were as follows: 95 °C, 3 min for initial denaturation, followed by 40 cycles of amplification (95 °C for 10 s and 60 °C for 30 s). The primers used in the experiment, listed in Table S2, were synthesized by Sigma-Genosys (The Woodlands, TX, USA). The relative expression levels of mRNA of target genes were normalized to GAPDH and calculated using the 2^−ΔΔCq^ method.

### 2.8. Statistical Analysis

All experiments were performed in triplicate unless otherwise specified. All data were presented as mean ± standard deviation (SD). One-way ANOVA (using the Tukey post hoc test) and two-way ANOVA (using the Tukey post hoc test) were calculated using GraphPad Prism 8 (GraphPad Software, Inc., La Jolla, CA, USA). Statistical significance was set at *p* < 0.05.

## 3. Results

### 3.1. Flazin Improved Cellular Lipid Content and Profile

#### 3.1.1. Cell Viability

**Figure 2:**
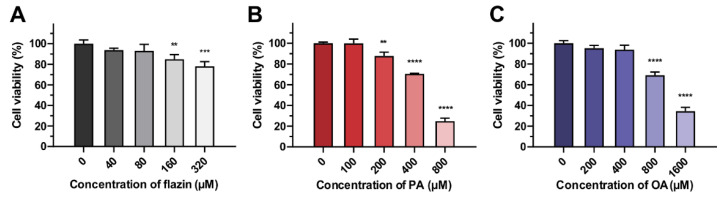
Viability of cells treated with flazin (**A**), palmitic acid (PA) (**B**), or oleic acid (OA) (**C**). ** *p* < 0.01, *** *p* < 0.001, **** *p* < 0.0001 vs. control (*n* = 3).

To determine the suitable dose for the following experiments, we firstly assessed the cytotoxicity of flazin on HK-2 cells. As shown in Figure 2A, flazin with a concentration lower than 80 μM exhibited no apparent influence on cell viability. Therefore, 40 μM and 80 μM of flazin were used to explore the function. At the same time, the appropriate concentrations of PA and OA were tested individually. As shown in Figure 2B, the increased concentration of PA caused a gradual decrease in cell viability, and a similar trend was also found in OA-treated groups (Figure 2C). The cell viabilities of 200 μM PA and 200 μM OA were 87.7% and 95.2%, respectively, which were selected in the following experiments to induce the fatty acid overloading in HK-2 cells for simulating the lipid burden during the progression of DN. Furthermore, we confirmed that the co-treatment of flazin and fatty acids did not cause extra cytotoxicity to cells (Figure S4).

#### 3.1.2. TG and FFA Content

**Figure 3:**
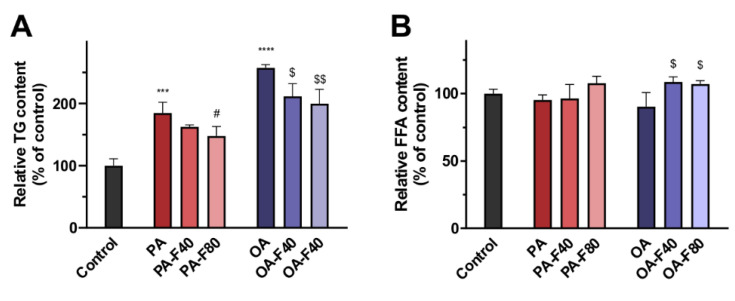
Effect of flazin on cellular lipids. (**A**) Cellular triglyceride (TG) content. (**B**) Cellular free fatty acid (FFA) content. PA, 200 μM palmitic acid; PA-F40, 200 μM palmitic acid + 40 μM flazin; PA-F80, 200 μM palmitic acid + 80 μM flazin; OA, 200 μM oleic acid; OA-F40, 200 μM oleic acid + 40 μM flazin; OA-F80, 200 μM oleic acid + 80 μM flazin. *** *p* < 0.001, **** *p* < 0.0001 vs. control, ^#^ *p* < 0.05 vs. PA group, ^$^ *p* < 0.05, ^$$^ *p* < 0.01 vs. OA group (*n* = 3).

LC/MS analysis was performed to investigate the effect of flazin on cellular TG and FFA content. Cells treated with PA or OA exhibited a drastic increase in total TG content compared with the control (1.9-fold and 2.6-fold, respectively), indicating them as in vitro models of lipid overloading in HK-2 cells. When co-treated with flazin, cellular TG levels were significantly reduced in a dose-dependent manner: the PA-F40 and the PA-F80 groups showed decreases in cellular TG levels by 12.0% ± 1.7% and 20.1% ± 8.3%, respectively (Figure 3A); in parallel, those of the OA-F40 and the OA-F80 groups were 17.9% ± 8.0% and 22.4% ± 8.9%, respectively.

Moreover, total FFA content was also analyzed and presented in Figure 3B. Interestingly, the addition of PA or OA had less effect on total FFA content, and comparatively, cells co-treated with flazin showed a modest increase in total FFA content.

#### 3.1.3. TG Profile and FFA Characteristics

**Figure 4:**
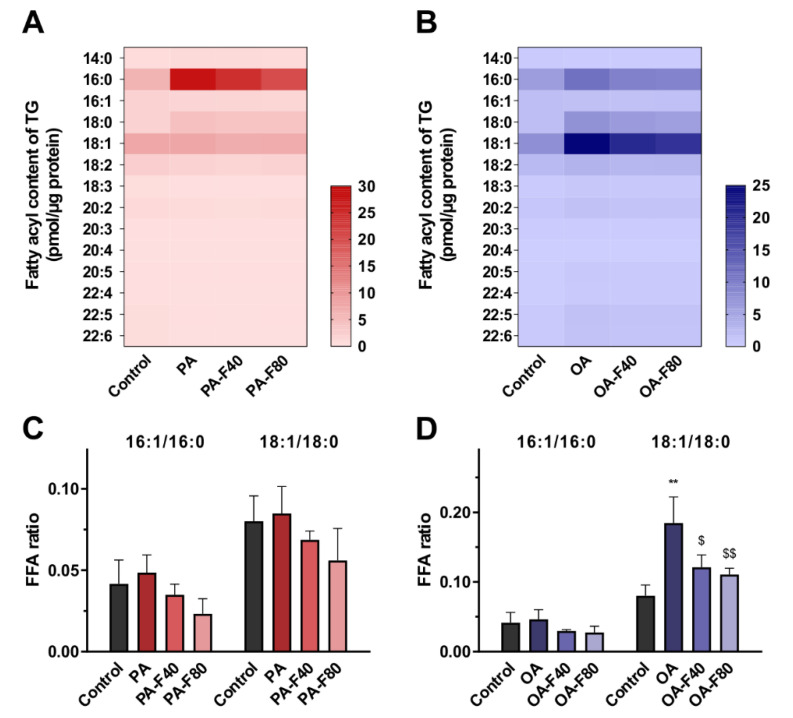
(**A**,**B**) Heatmap of fatty acyl composition of TG in the cells treated with PA (**A**) or OA (**B**). (**C**,**D**) FFA ratio of 16:1 to 16:0 and 18:1 to 18:0 in the groups treated with PA (**C**) or OA (**D**). PA, 200 μM palmitic acid; PA-F40, 200 μM palmitic acid + 40 μM flazin; PA-F80, 200 μM palmitic acid + 80 μM flazin; OA, 200 μM oleic acid; OA-F40, 200 μM oleic acid + 40 μM flazin; OA-F80, 200 μM oleic acid + 80 μM flazin. ** *p* < 0.01 vs. control, ^$^ *p* < 0.05, ^$$^ *p* < 0.01 vs. OA group (*n* = 3).

The fatty acyl composition of TG was elucidated according to the MS/MS fragmentation. The heatmaps indicated the alteration of fatty acyl composition (Figure 4A,B): the PA group showed a drastic increase in fatty acyl 16:0. In comparison, fatty acyl 16:0 levels in the PA-F40 and the PA-F80 groups were decreased by 13.4% ± 4.1% and 28.0% ± 8.6%, respectively (Figure 4A). In addition, fatty acyl 16:0, 18:0, and 18:1 increased in the OA group, but they were largely decreased by 19.9% ± 9.1%, 18.7% ± 7.7%, and 17.0% ± 7.5% in the OA-F80 group, respectively (Figure 4B).

In addition, the content of individual FFA molecules was also determined. The ratios of 16:1/16:0 and 18:1/18:0 can be utilized as the indicator of SCD-1 activity, also known as the SCD index [26]. As expressed in Figure 4C,D, SCD indexes were elevated when treated with PA or OA, especially for 18:1/18:0 of the OA group, which accounted for 2.3-fold of the control. When flazin was added, the SCD index showed a declined trend.

### 3.2. Flazin Alleviated LD Accretion

**Figure 5:**
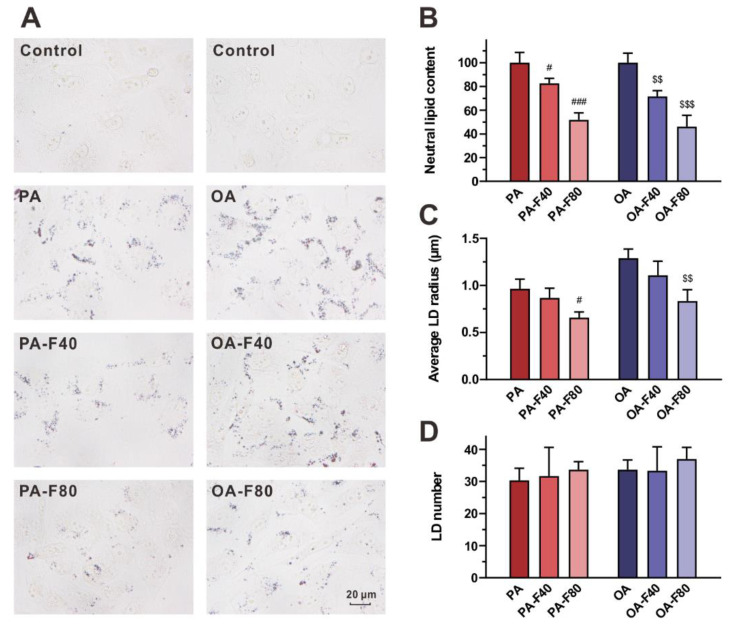
Effect of flazin on lipid droplet (LD) accumulation. (**A**) Representative photographs of oil red O staining. (**B**) Neutral lipid content. PA, PA-F40 and PA-F80 groups are presented as relative amount to PA group; OA, OA-F40 and OA-F80 groups are presented as relative amount to OA group. (**C**) Average LD size. (**D**) LD number. PA, 200 μM palmitic acid; PA-F40, 200 μM palmitic acid + 40 μM flazin; PA-F80, 200 μM palmitic acid + 80 μM flazin; OA, 200 μM oleic acid; OA-F40, 200 μM oleic acid + 40 μM flazin; OA-F80, 200 μM oleic acid + 80 μM flazin. ^#^ *p* < 0.05, ^###^ *p* < 0.001 vs. PA group, ^$$^ *p* < 0.01, ^$$$^ *p* < 0.001 vs. OA group (*n* = 3).

Oil red O staining was performed to visualize lipid deposition in LDs and evaluate the effect of flazin on LD accumulation. As shown in Figure 5A, the redly stained particles indicated the accumulation of neutral lipids in LDs of the lipid-overloaded cells. In the control group, LDs were hardly accumulated, so they could not be clearly examined or accurately calculated. In the PA and OA groups, LDs were obviously accumulated. Compared with the modeling groups, flazin treatment evidently decreased lipid deposition dose-dependently (Figure 5A).

The neutral lipid content, the average size of LDs, and the LD number were also calculated and are shown in Figure 5B–D, respectively. Neutral lipid content was greatly reduced with flazin treatment compared to the PA and the OA groups (Figure 5B): the levels in the PA-F40 and the PA-F80 groups were 82.6% ± 4.1% and 51.8% ± 5.9% of the PA group, respectively, and the levels in the OA-F40 and the OA-F80 groups were as low as 71.6% ± 4.9% and 46.1% ± 9.6% of the OA group, respectively (*p* < 0.05 for all).

Moreover, flazin also decreased the LD size (Figure 5C); in particular, 80 μM of flazin exhibited a significant downsizing effect upon the LD size by 31.9% ± 6.4% and 35.3% ± 9.3% compared to the PA and OA groups, respectively (*p* < 0.05 for both). The LD number showed negligible change with different treatments.

### 3.3. Flazin Reduced TG Level in LDs

**Figure 6:**
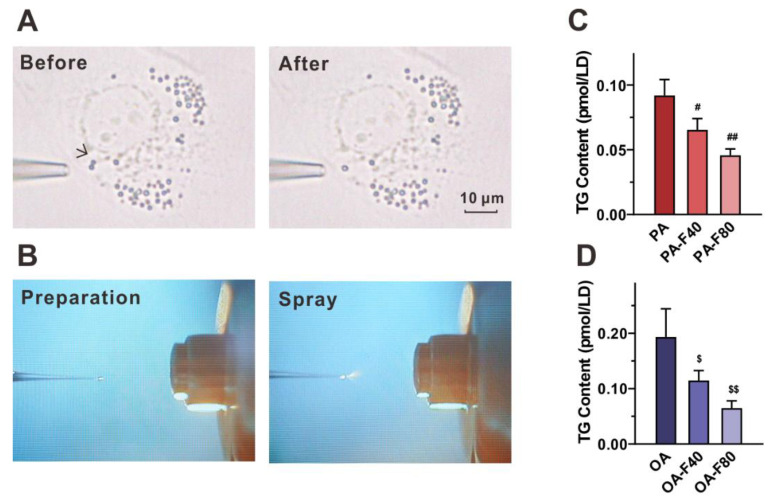
Effect of flazin on LD TG content. (**A**) Representative photographs of LDs before and after aspiration. (**B**) Microscopic inspection on the tip of nanoESI emitter. (**C**,**D**) LD TG content in the cells treated with PA (**C**) or OA (**D**). PA, 200 μM palmitic acid; PA-F40, 200 μM palmitic acid + 40 μM flazin; PA-F80, 200 μM palmitic acid + 80 μM flazin; OA, 200 μM oleic acid; OA-F40, 200 μM oleic acid + 40 μM flazin; OA-F80, 200 μM oleic acid + 80 μM flazin. ^#^ *p* < 0.05, ^##^ *p* < 0.01 vs. PA group, ^$^ *p* < 0.05, ^$$^ *p* < 0.01 vs. OA group (*n* = 3).

To directly investigate the lipids deposited in LDs, we aspirated the single LDs from the cells in situ using nanotips (Figure 6A) and performed the in-tip solvent microextraction, followed by nanoESI-MS analysis (Figure 6B). The inducement of PA or OA resulted in the LD TG content of 0.09 ± 0.01 pmol/LD and 0.19 ± 0.05 pmol/LD, respectively. Supplementation of flazin reversed such accretion effectively: the PA-F40 and the PA-F80 groups significantly decreased the LD TG content by 28.9% ± 9.5% (*p* < 0.05 vs. PA group) and 50.2% ± 5.3% (*p* < 0.01 vs. PA group), respectively (Figure 6C); the OA-F40 and the OA-F80 groups exhibited an even stronger potency, decreasing by 40.7% ± 9.3% (*p* < 0.05 vs. OA group) and 66.4% ± 6.7% (*p* < 0.01 vs. OA group), respectively (Figure 6D).

### 3.4. Flazin Regulate Surface Properties of LDs

**Figure 7:**
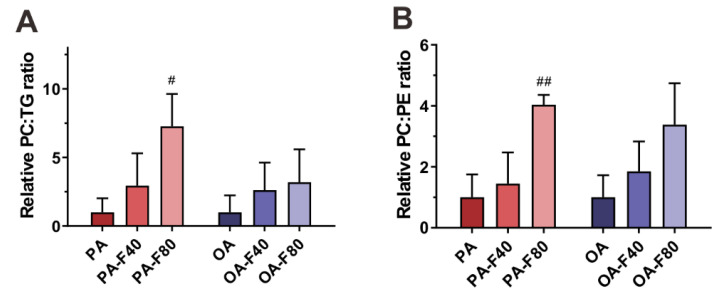
Effect of flazin on LD surface properties. (**A**) Relative phosphatidylcholine–triglyceride (PC–TG) ratio of LD in the cells treated with PA or OA. (**B**) Relative phosphatidylcholine–phosphatidylethanolamine (PC–PE) ratio of LD in the cells treated with PA or OA. PA, 200 μM palmitic acid; PA-F40, 200 μM palmitic acid + 40 μM flazin; PA-F80, 200 μM palmitic acid + 80 μM flazin; OA, 200 μM oleic acid; OA-F40, 200 μM oleic acid + 40 μM flazin; OA-F80, 200 μM oleic acid + 80 μM flazin. ^#^ *p* < 0.05, ^##^ *p* < 0.01 vs. PA group (*n* = 3).

As the membrane composition, PC and PE were also investigated because of their crucial role in regulating LD morphology and biophysical properties. The relative content of PC to TG represents PC availability to LDs. As shown in Figure 7A, the PC–TG ratio exhibited an increasing trend with the supplement of flazin, especially 80 µM of flazin which increased the PC–TG ratio by 6.3-fold compared with the PA group.

Furthermore, the relative content of PC to PE was also investigated as a factor associated with packing defects [27]. As shown in Figure 7B, flazin increased the PC–PE ratio, especially at 80 μM (by 3.0- and 2.4-fold of the PA and the OA groups, respectively).

### 3.5. Flazin Regulated mRNA Expression of Lipogenic and Lipolytic Genes

**Figure 8:**
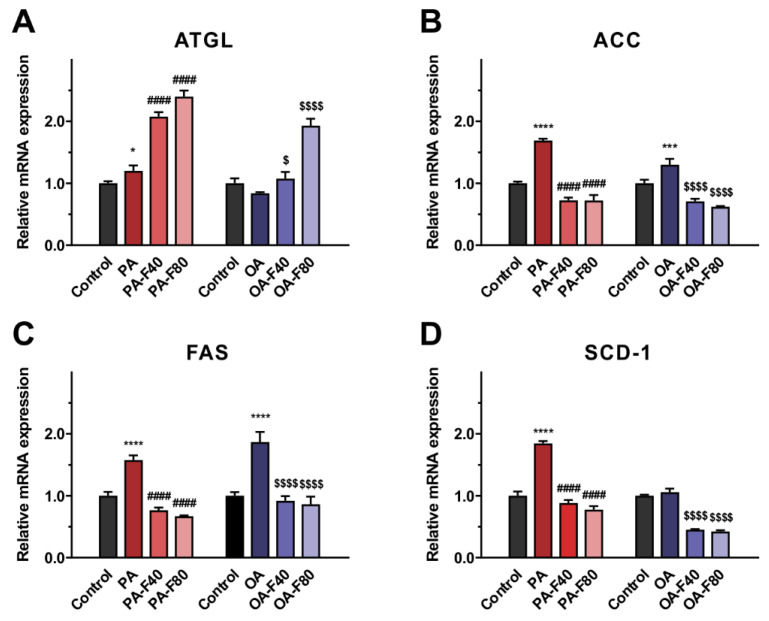
Effect of flazin on mRNA expression of adipose triglyceride lipase (ATGL) (**A**), acetyl-CoA carboxylase (ACC) (**B**), fatty acid synthase (FAS) (**C**), and stearoyl-CoA desaturase-1 (SCD-1) (**D**). PA, 200 μM palmitic acid; PA-F40, 200 μM palmitic acid + 40 μM flazin; PA-F80, 200 μM palmitic acid + 80 μM flazin; OA, 200 μM oleic acid; OA-F40, 200 μM oleic acid + 40 μM flazin; OA-F80, 200 μM oleic acid + 80 μM flazin. * *p* < 0.05, *** *p* < 0.001, **** *p* < 0.0001 vs. control, ^####^ *p* < 0.0001 vs. PA group, ^$^ *p* < 0.05, ^$$$$^ *p* < 0.0001 vs. OA group (*n* = 3).

To explore the underlying mechanisms for the regulating effect of flazin on lipid content and profile, we also investigated the expression of lipolytic- and lipogenic-related genes. ATGL serves as a rate-limiting enzyme that catalyzes the initial TG hydrolysis in LDs and plays a critical role in energy homeostasis [28]. Flazin treatment markedly upregulated the ATGL mRNA expression compared to the PA or the OA groups (*p* < 0.05 for all the flazin-treated groups, shown in Figure 8A). In addition, ACC, FAS, and SCD-1 are the primary enzymes modulating fatty acid synthesis [29], all of which the mRNA expression was upregulated with PA or OA intervention (except for SCD-1 in the OA group). Importantly, when co-treated with flazin, the mRNA expression levels of the three genes were downregulated to approximately half of that in the PA or the OA group (*p* < 0.0001 for all, Figure 8B–D). Interestingly, no dose-dependent behavior was found in these fatty acid synthesis-related genes, indicating that flazin exhibited a prominent suppressive effect on lipogenesis at a relatively low dose (40 µM).

## 4. Discussion

Lipid-storage disorders, such as ELA, disrupt lipid homeostasis and cause a series of serious metabolic diseases. ELA in the tubular portion of the kidney induces lipotoxicity, which is considered a novel mechanism for the progression of DN [8]. Recent studies suggested that the management of lipid metabolism contributes to the treatment of this diabetic complication [30,31]. Food-derived bioactive components have long been used as a complementary therapy to promote healthcare and treat kidney diseases due to their low treatment costs and minimal side effects. Hence, this study investigated the effect of flazin in lipid metabolism at both cell and organelle levels.

The LC/MS analysis of cellular TG and FFA proved a promising lipid-lowering effect of flazin in HK-2 cells. In both PA and OA treated groups, flazin significantly decreased total TG content and modestly increased total FFA content. Cells respond to the elevated FFA level by esterifying FFA to TG and storing it in LDs [32]. Non-adipocytes are poorly adapted to store excess TG and may suffer from lipotoxicity. According to the current results, flazin remarkably suppressed TG deposition, and at the same time, showed a negligible effect on FFA content, which suggested that flazin could inhibit TG accumulation in HK-2 cells and thus prevent the potential damages caused by ELA.

MS/MS fragmentation data indicated that flazin improved TG profile and FFA characteristics against lipid disorders. Specifically, flazin ameliorated the alteration of TG fatty acyl composition caused by fat overloading. Additionally, flazin modified relative content of FFA 16:0, 16:1, 18:0, and 18:1. FFA 16:0 and 18:0 can be dehydrogenated by SCD-1 to form FFA 16:1 and 18:1. The ratios of 16:1/16:0 and 18:1/18:0 can be utilized as the indicator of SCD-1 activity, known as the SCD index. The deficient SCD-1 activity was reported to contribute to reducing body adiposity, increasing insulin sensitivity, and alleviating diet-induced obesity [33]. In our study, treatment with flazin was found to reduce SCD index against PA- or OA-induced FFA dysregulation, which helped ameliorate fat accumulation in HK-2 cells.

In addition to the lipid-lowering effect in cells, flazin exhibited a multi-dimension regulatory effect on LD morphology. Flazin exhibited a down-sizing effect on LDs, which was revealed by oil red O staining. It is reported that small LDs facilitate lipolysis by providing a larger surface area for lipases, while those LDs with a larger size contribute to insufficient lipolysis, leading to the disturbance of TG metabolism, lipid utilization, and energy production [34]. Flazin markedly decreased LDs size of cells with lipid overloading, which contributed to preventing the formation of supersized LDs and helped maintain TG metabolism. It should be noted that, as oil red O can stain all neutral lipid to be red, there were some differences between results from LC/MS and oil red O staining. Another limitation is the resolution of the microscopic technique employed in the experiment: those very small LDs beyond the resolution limit might be ignored. Nevertheless, when excess fatty acids were loaded, the TG level increased rapidly, and large LDs were formed (Figure 5A). Compared to cells only treated with PA or OA, flazin treatment significantly decreased LD size. Therefore, this limitation did not interfere with the comparison between the model group and treatment group.

In addition, flazin modulates LDs core by decreasing TG level. Oil red O staining indicated that flazin markedly decreased neutral lipid content in LDs in a dose-dependent manner. LD number was determined, and negligible change was found in cells with different treatments. The lower TG content (Figure 5B) was in agreement with the smaller LD size (Figure 5C). The TG-lowering effect of flazin on LDs was also proved by NanoESI-MS. It should be noticed that the LD TG-lowering effect of flazin (Figure 6C,D) was even greater than that on cellular TG (Figure 3A), indicating a selective inhibition targeting LDs. In non-adipocytes, although TG is necessary for various physiological activities, excess TG molecules are packed into LDs, resulting in ELA and contributing to lipotoxicity. Therefore, the preferential hydrolysis of LD TG is of great benefit. Direct analysis of LD TG by nanoESI-MS proved this effect of flazin.

**Figure 9:**
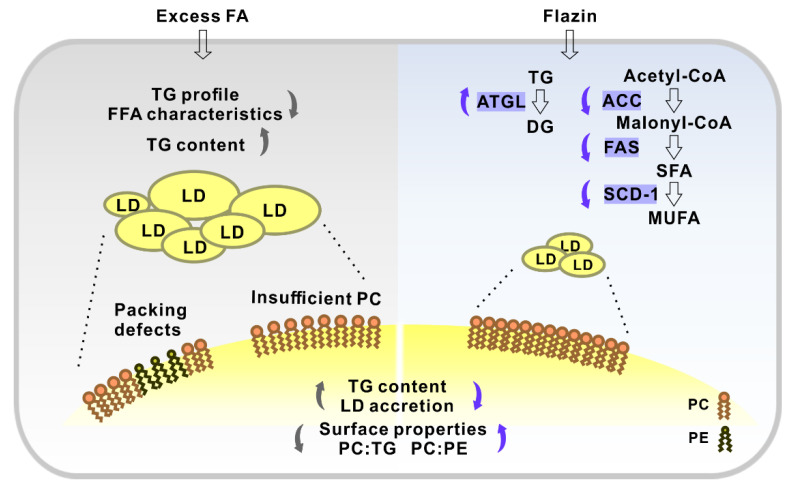
Schematic illustration showing the effect of flazin on cellular lipid and lipid droplets (LDs). FA, fatty acid; SFA, saturated fatty acid; MUFA, monounsaturated fatty acid; TG, triglyceride; DG, diglycerol; PC, phosphatidylcholine; PE, phosphatidylethanolamine; ATGL, adipose triglyceride lipase; ACC, acetyl-CoA carboxylase; FAS, fatty acid synthase; SCD-1, stearoyl-CoA desaturase-1.

Moreover, flazin regulates LD surface properties by modifying the relative content of TG, PC, and PE. It is known that PC acts as a surfactant to shield underlying TG from surrounding cytosol and prevent LD coalescence, and the relative content of PC to TG represents the PC availability to LDs [35,36]. When PC is insufficient, the hydrophobic LD core is exposed to the surrounding aqueous environment, and fusion is induced to decrease the surface-to-volume ratio of LDs, causing the formation of supersized LDs (Figure 9). Therefore, our current results that flazin increased the PC–TG ratio were in line with its preventive effect against the coalescence and the formation of supersized LDs. However, as the surface area-to-volume ratio of a sphere increased along with the decreasing radius, in the present experiments, how the increasing PC–TG ratio matches the reducing LD size remained unknown, which needed further molecular monitoring investigation. In addition, we also found that flazin increased the PC–PE ratio compared with the modeling groups. PC–PE ratio adversely affects membrane integrity and thus was suggested as a factor associated with packing defects [27], which meant the interfacial voids at the membrane-water interface could accommodate the binding of hydrophobic residues [37]. In biological membranes, PC forms cylindrical structures, while PEs form conical structures [38]. Therefore, a decreased PC–PE ratio is considered to promote packing defects and thereby favor the localization of perilipin to LD surface, which contributes to lipid accumulation and LDs growth [27]. According to the present data, flazin increased the PC–PE ratio, which suggested its reducing effect on packing defects and thereby preventing LD enlargement (Figure 9). It should also be noted that domain size characterization over a wide range of molar proportions of PC and PE may be necessary for further investigation on the packing defects to prove the effects. Taken together, these results suggested that flazin could regulate LD surface properties by modifying the LD membrane phospholipid composition, thereby potentially preventing the fusion and enlargement of LDs. Nevertheless, molecular dynamic simulation is needed for further confirmation and elucidation.

Finally, the mRNA expression of lipogenic and lipolytic genes was tested to clarify the potential mechanism of the lipid-lowering effect of flazin. In the present study, flazin treatment upregulated ATGL expression and downregulated ACC, FAS, and SCD-1. The elevated ATGL expression is believed to promote TG hydrolysis, resulting in a reduced TG content [39], which was proved by LC/MS-based cellular lipidomic analysis (Figure 3A). The lowered expression of ACC, FAS, and SCD-1 indicated the suppression of fatty acid synthesis by flazin treatment. In particular, the SCD-1 downregulation (Figure 8D) was in agreement with the decreased SCD indexes (Figure 4C,D), indicating the consistency of gene expression and lipid metabolism regulated by flazin. It should also be noted if there are other potential mechanisms accounting for the effect of flazin, such as decreased intake and mitochondrial consumption. Further investigation is needed to clarify this hypothesis. In short, flazin contributed to a reduction in TG content in HK-2 cells. The mechanisms associated need more experiments to gain understanding.

## 5. Conclusions

To conclude, this study discovered the improving effects of flazin on lipid metabolism at the cell level and organelle level. Flazin lowered cellular TG content, modulated TG profile, and improved FFA characteristics. Moreover, flazin exhibited a multi-dimension regulatory effect on LDs. Flazin alleviated LD accretion, decreased LD TG level, and improved LD morphology, including LD size and membrane properties. The potential mechanisms might be the inhibition of lipogenesis and the promotion of lipolysis. These findings suggested that flazin served as a promising nutraceutical candidate for DN treatment by improving lipid metabolism and regulating LD dynamics. Furthermore, this study proposed a strategy combining biological assays and chemical analyses covering both cells and organelles, which may contribute to discovering and evaluating other nutraceuticals and nutrition supplements for preventing or treating metabolic diseases related to lipid disorders and lipid-storage disorders.

## Data Availability

Data is contained within the article or Supplementary Materials.

## Supplementary Materials

The following supporting information can be downloaded at: https://www.mdpi.com/article/10.3390/nu14071501/s1, Figure S1: Synthetic route of flazin; Figure S2: ^1^H NMR and ^13^C NMR spectra of the prepared flazin; Figure S3: ESI-MS of the prepared flazin; Figure S4: Viabilities of cells co-treated with flazin and fatty acids; Table S1: Content of TG and FFA species by LC/MS; Table S2: Sequence of primers for quantitative real-time PCR.
