# Supplementary material for: Flazin as a Lipid Droplet Regulator against Lipid Disorders
Source: Nutrients. 2022 Apr 3;14(7):1501. doi: 10.3390/nu14071501 (PMC9002757; doi:10.3390/nu14071501)
Supplement: Supplementary file 1 [file nutrients-14-01501-s001.zip › nutrients-1618620-supplementary.pdf]

Supplementary Material

Preparation of flazin by chemical synthesis

For the synthesis of flazin, 2.18 mL of $SOCl_2$ was added into 50 mL of methanol containing 10 mmol of L-tryptophan, and the solution was stirred overnight. The pH of the solution was adjusted to 9–10 by adding NaOH aqueous solution, and the solution was extracted with ethyl acetate. Organic layers were collected and evaporated to give compound 2. Then, 1 mmol of compound 2 was added into 10 mL of CH_2Cl_2 containing 1 mmol of 5-(hydroxymethyl)furfural and 80 μL of trifluoroacetate (TFA). The mixture was stirred for 3 h and then neutralized with triethylamine (TEA). Another 0.5 mL of TEA and 1 mmol of trichloroisocyanuric acid was added, and the mixture was stirred for 2 h. The solution was extracted with ethyl acetate. Organic layers were collected and evaporated to give compound 4. Next, compound 4 (1 mmol) was dissolved in 5 mL of methanol, and NaOH aqueous solution (3 mol/L, 1 mL) was added. The mixture was heated to 65 °C and stirred for 5 h. Afterward, citric acid was added to adjust the pH to 4–5. Then, the solution was extracted with ethyl acetate. The collected organic layer was purified using flash chromatography to yield the final product (flazin).

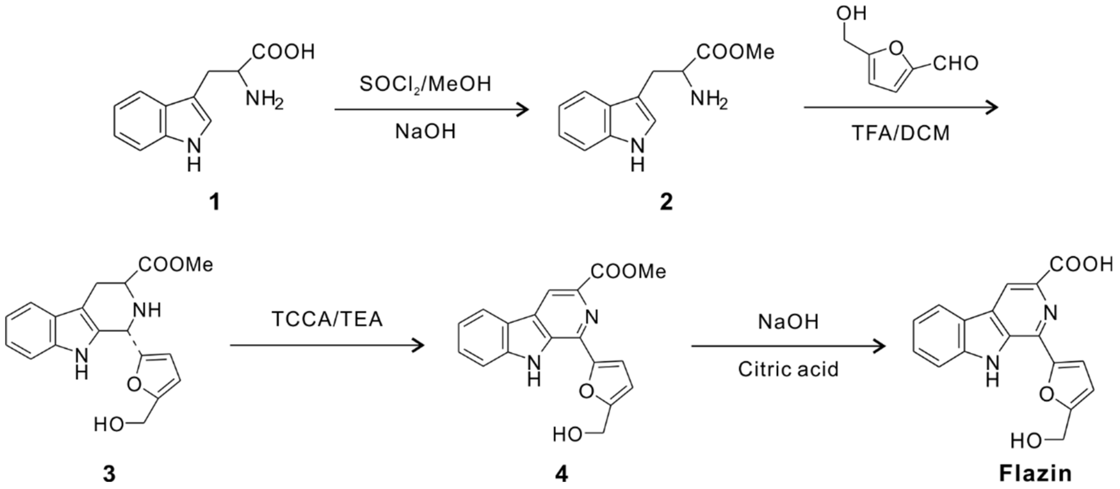

Figure S1. Synthetic route of flazin.

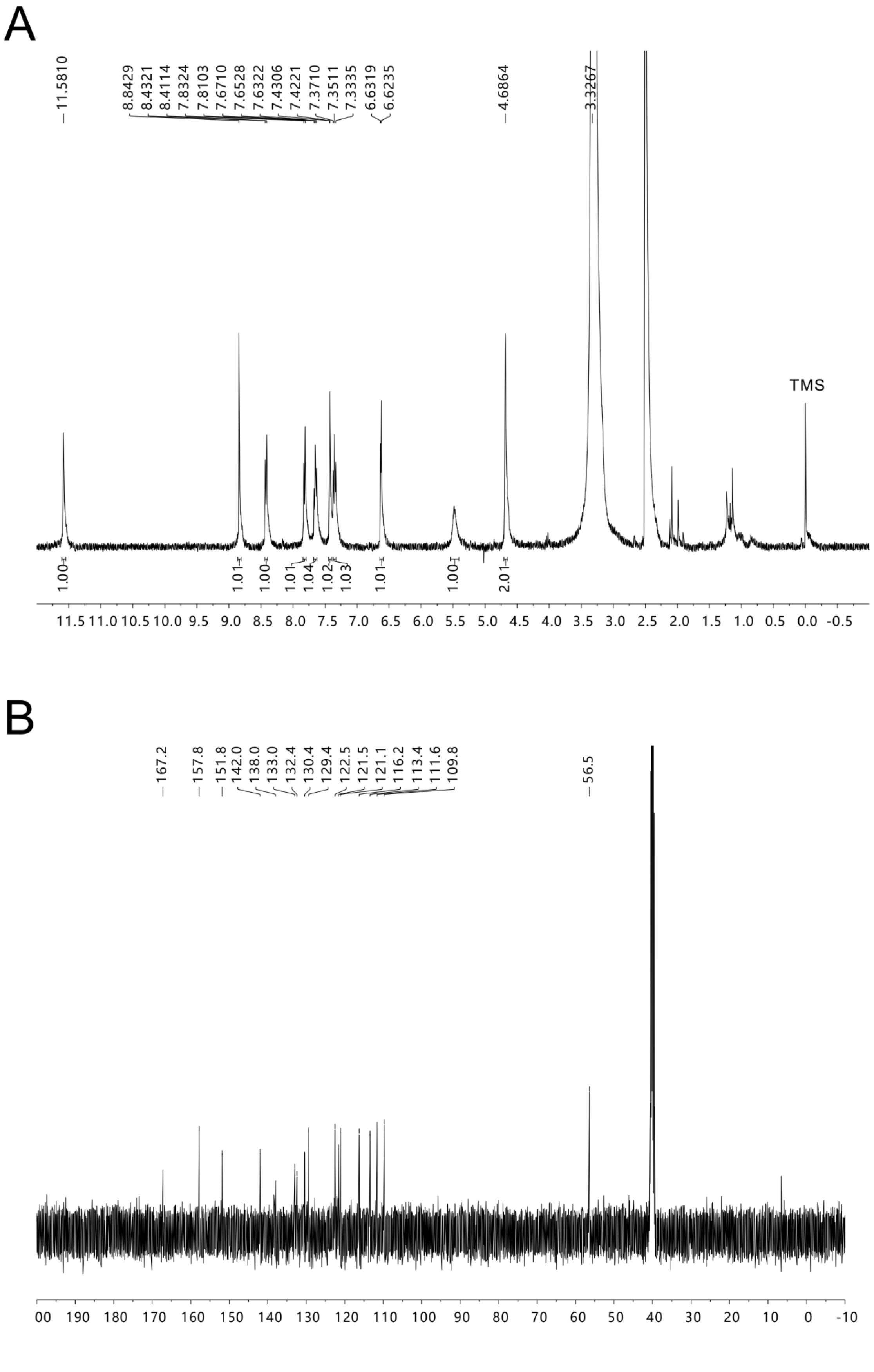

Figure S2. (A) ^{1}H NMR spectrum and (B) ^{13}C NMR of the prepared flazin.

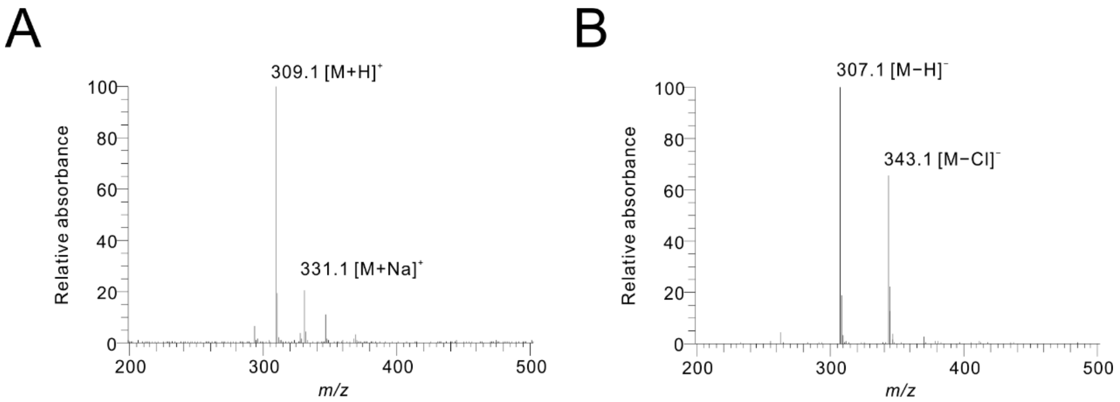

Figure S3. ESI-MS of the prepared flazin in (A) positive mode and (B) negative mode.

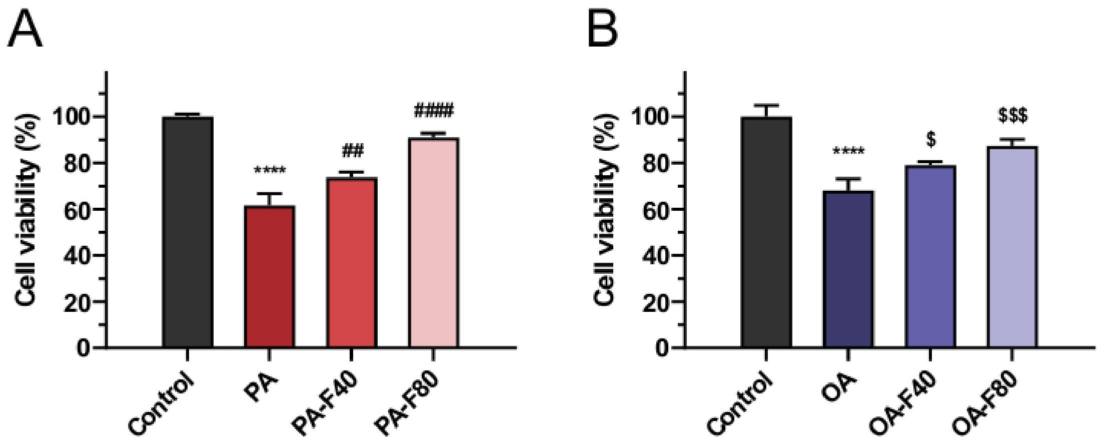

Figure S4. Viabilities of cells treated with flazin and fatty acids. PA, 400 μM palmitic acid; PA-F40, 400 μM palmitic acid + 40 μM flazin; PA-F80, 400 μM palmitic acid + 80 μM flazin; OA, 800 μM oleic acid; OA-F40, 800 μM oleic acid + 40 μM flazin; OA-F80, 800 μM oleic acid + 80 μM flazin. **** $p < 0.0001$ vs. control, ## $p < 0.01$, #### $p < 0.0001$ vs. PA group, \$ $p < 0.05$, \$\$\$ $p < 0.001$ vs. OA group (n = 3).

Table S1. Content of TG and FFA species by LC/MS (means ± SD, pmol/mg protein).

Lipid species	Control	PA	PA-F40	PA-F80	OA	OA-F40	OA-F80
TG							
42:0	31.2±7.9	23.6±4.7	30.1±17.6	25.9±19.6	22.8±18.1	22.0±7.8	24.2±5.7
44:0	72.6±13.5	89.3±9.9	99.9±33.2	91.4±40.7	51.4±31.2	51.6±17.8	51.6±9.6
46:0	73.9±13.1	346.8±33.6	331.3±28.8	280.1±74.7	45.4±22.6	46.8±15.0	43.9±5.1
46:1	219.7±37.9	233.6±28.4	249.5±84.7	221.2±87.0	151.4±100.6	139.7±49.0	155.3±36.9
46:2	145.8±28.5	105.8±19.8	117.8±56.0	109.0±61.0	100.5±72.6	95.2±35.3	106.5±22.6
46:3	37.0±7.7	26.0±4.2	27.8±12.5	24.3±13.2	23.4 ±15.6	23.9±8.4	26.5±6.0
48:0	133.3±14.4	4080.7±211.8	3448.5±282.7	2574.7±326.6	78.1±17.7	63.3±13.7	61.0±6.4
48:1	150.6±12.9	752.3±112.7	595.2±58.0	494.3±104.9	101.8±40.0	92.4±27.9	99.3±13.6
48:2	235.5±30.8	254.2±28.9	247.8±67.3	230.9±84.4	172.9±85.9	159.9±52.3	174.6±29.7
48:3	98.4±14.2	71.3±11.4	77.4±32.0	70.5±33.2	68.4±42.6	65.4±23.5	71.9±13.9
48:4	16.4±3.0	12.6±2.1	13.3±2.5	11.7±4.3	12.0±6.0	11.1±3.8	12.6±2.9
48:5	4.3±2.9	1.9 ±1.8	1.8±1.2	1.6±1.2	1.8±1.4	1.1±0.8	1.6±0.9
50:0	75.4±6.8	1510.4±109.2	1358.4±121.0	1155.0±202.8	38.9±1.8	39.8±6.5	42.2±6.8
50:1	494.0±60.6	2202.5±226.8	1894.2±97.7	1616.6±158.9	226.0±26.6	211.2±35.9	230.7±28.3
50:2	300.7±36.9	426.8±58.9	384.0±43.5	374.9±54.5	396.4±18.8	365.5±65.4	383.6±69.7
50:3	137.1±17.3	98.6±13.2	97.8±26.3	99.0±30.8	127.6±25.3	110.5±20.0	120.5±14.5
50:4	56.0±5.3	38.3±6.7	36.4±9.4	35.3±9.5	43.8±10.5	38.9±8.2	40.2±4.4
50:5	14.5±2.9	8.1±2.3	6.5±1.0	6.8±1.3	12.0±1.0	9.9±1.7	9.9±1.9
50:6	2.5±0.8	0.3±0.2	0.4±0.3	0.3±0.2	1.2±0.1	1.0±0.2	1.0±0.2
52:0	49.4±3.9	284.7±12.1	251.0±26.0	230.1±28.0	51.7±33.9	97.2±6.7	98.5±9.7
52:1	300.5±32.0	797.3±89.6	756.0±65.1	714.8±71.8	166.3±29.3	152.4±31.7	175.4±45.9
52:2	761.2±76.7	689.1±66.0	650.6±30.2	622.0±20.6	1520.1±46.1	1510.4±102.3	1538.6±104.9
52:3	456.5±40.3	348.8±42.8	287.8±33.1	300.1±17.7	1397.0±90.0	1135.9±101.2	1074.2±144.0
52:4	250.8±20.0	224.9±126.5	237.3±23.8	277.8±11.4	287.9±67.5	233.1±64.3	256.0±122.1
52:5	125.5±7.8	113.5±26.1	93.3±7.3	113.3±2.2	183.9±8.3	147.2±29.1	144.4±47.0
52:6	36.7±10.5	21.2±6.5	17.1±1.8	19.7±1.8	39.6±2.4	31.6±5.4	31.3±7.8
52:7	6.0±2.6	0.8±0.5	0.5±0.3	0.6±0.4	2.3±0.2	1.5±0.1	2.0±0.5
52:8	11.2±1.0	13.6±3.8	12.2±1.0	6.9±2.7	15.0±1.7	10.0±1.0	9.2±1.5
54:0	38.8±3.2	78.0±1.3	78.6±3.8	77.5±10.3	65.9±57.9	149.0±7.3	152.7±11.4
54:1	80.4±12.4	171.6±19.7	165.7±12.7	182.4±37.4	50.4±8.5	45.9±10.2	53.2±10.7
54:2	311.8±59.8	233.7±28.7	211.7±16.2	244.9±30.2	1435.0±114.3	1259.3±109.3	1120.7±84.8
54:3	495.8±51.0	315.9±26.2	286.4±27.1	394.1±106.7	8476.0±541.4	6288.0±379.4	5291.4±355.7
54:4	451.2±27.1	342.8±33.6	260.0±108.7	369.3±29.6	841.8±23.8	688.9±63.3	666.1±105.9
54:5	373.6±15.1	391.4±82.2	284.8±21.9	258.3±50.9	412.6±65.1	349.3±86.8	374.5±144.5
54:6	442.7±37.0	316.1±51.6	261.7±29.1	304.2±87.8	452.4±137.7	360.3±122.1	388.4±203.9
54:7	174.1±10.5	89.8±14.5	73.2±9.7	118.1±33.5	166.3±55.7	129.8±48.4	147.5±91.2
54:8	27.4±0.6	10.0±1.1	9.4±1.4	15.6±5.1	25.1±8.1	19.2±7.2	22.5±13.9
54:9	2.6±0.4	0.2±0.2	0.1±0.1	0.7±0.3	2.4±0.3	1.8±0.6	2.0±0.9
56:4	140.4±41.4	89.4±20.9	84.0±8.6	93.3±17.8	315.6±20.2	278.4±23.0	281.8±23.0
56:5	254.2±87.1	167.6±38.6	153.8±16.2	158.8±36.9	514.6±43.8	427.2±20.1	401.2±27.5

Lipid Species	Control	PA	PA-F40	PA-F80	OA	OA-F40	OA-F80
56:6	285.2±105.4	96.9±27.2	84.8±9.1	93.0±20.8	784.5±102.6	637.7±62.7	633.6±98.3

56:7	150.1±58.7	37.6±13.2	30.9±3.5	35.6±8.7	283.9±42.4	227.1±27.2	235.0±44.0
56:8	55.6±24.3	10.5±4.4	8.9±1.5	10.1±2.7	60.4±11.2	47.0±4.9	51.7±8.7
56:9	14.9±6.7	3.4±1.7	2.6±0.5	2.8±0.8	10.1±2.0	7.9±1.0	9.0±1.8
56:10	3.5±2.0	0.4±0.4	0.3±0.1	0.3±0.2	2.8±0.6	2.0±0.3	2.3±0.7
56:11	0.3±0.2	0.0 ±0.0	0.0±0.0	0.0±0.0	0.1±0.1	0.0±0.0	0.0±0.0
58:6	187.0±68.4	43.4±12.1	40.3±5.5	47.9±11.7	501.9±48.9	442.9±32.8	460.8±38.8
58:7	177.5±67.0	19.1±6.6	17.6±1.9	23.6±7.4	831.4±91.0	683.3±53.3	673.7±99.0
58:8	99.9±39.8	13.0±5.4	10.7±1.8	14.3±4.4	500.4±65.5	400.3±44.9	389.5±67.3
58:9	47.5±20.6	10.0±4.7	8.0±1.1	9.8±3.2	42.7±7.1	32.3±3.9	35.6±7.0
58:10	26.1±11.7	8.7±4.0	6.7±1.2	7.8±1.9	17.9±4.2	13.7±1.8	15.0±2.2
58:11	7.9±3.8	2.1±1.1	1.7±0.3	1.6±0.5	3.9±0.9	3.0±0.5	3.8±1.0
58:12	1.0±0.6	0.0±0.0	0.0±0.0	0.0±0.0	0.2±0.1	0.1±0.1	0.2±0.1
60:10	45.1±18.8	7.2±5.4	6.4±1.0	7.6±2.0	46.1±8.0	36.1±4.9	39.6±8.1
60:11	32.6±14.5	6.7±3.3	5.1±0.9	6.0±1.6	30.3±5.8	23.3±3.2	26.5±4.9
60:12	12.0±5.4	2.7±1.3	1.9±0.5	2.2±0.6	8.2±1.9	6.4±0.8	7.0±1.7
60:13	1.5±0.8	0.0±0.0	0.0±0.0	0.0±0.0	0.5±0.3	0.2±0.1	0.5±0.1
60:14	0.1±0.0	0.0±0.1	0.0±0.0	0.1±0.1	0.1±0.0	0.1±0.1	0.2±0.1
62:12	16.1±7.6	0.3±0.3	0.1±0.1	0.2±0.1	19.7±3.7	14.7±2.5	16.8±3.4
62:13	7.4±3.8	0.3±0.4	0.1±0.0	0.2±0.1	7.5±1.9	5.3±0.9	6.5±1.4
62:14	1.1±0.7	0.0±0.0	0.0±0.0	0.0±0.0	0.3±0.2	0.1±0.1	0.5±0.2
FFA							
14:0	2357.3±209.9	2192.6±136.8	2270.2±192.3	2358.2±263.1	2644.3±712.9	2492.6±164.7	2356.1±177.3
14:1	96.8±55.0	89.8±25.8	62.8±5.6	65.4±19.5	61.1±16.2	55.5±4.4	59.8±31.6
16:0	18825.1±549.0	18409.8± 593.2	18492.3±2697.5	22532.3±1430.6	13511.3±1263.3	19891.6±1689.5	20752.8±2303.5
16:1	787.0±292.7	896.2±226.7	638.8±89.7	515.8±170.3	623.3±188.4	596.3±76.9	574.9±225.4
16:2	11.1±5.3	9.4±2.6	6.7±1.6	6.9±3.3	6.9±2.4	7.9±1.4	7.1±2.8
18:0	24023.1±977.0	22637.5±1255.6	23549.4±2755.2	25729.6±1665.5	23122.2±4510.5	26180.4±2597.8	25890.0±1493.0
18:1	1917.6±319.1	1926.6±412.1	1622.6±303.8	1428.3±444.5	4162.5±310.6	3144.6±167.0	2861.6±262.1
18:2	1237.2±237.3	887.9±267.6	978.7±255.9	862.3±155.2	447.2±371.8	1250.5±386.7	678.2±144.3
18:3	104.3±22.4	74.7±25.2	76.7±26.0	65.0±13.1	56.5±18.7	102.5±33.8	53.2±11.1
20:0	1200.9±100.7	918.7±141.1	980.8±222.8	892.5±66.0	808.2±122.1	1184.4±227.1	840.0±104.3
20:1	117.2±27.2	99.3±28.0	91.4±18.0	85.5±29.4	208.4±12.6	175.2±15.0	176.2±7.5
20:2	50.1±21.7	42.5±10.5	37.3±9.3	34.6±18.1	37.2±14.8	36.0±5.1	46.1±13.5
20:3	42.0±3.6	65.2±22.1	57.6±17.8	64.7±23.9	45.1±3.9	34.8±2.4	43.2±5.9
20:4	130.6±49.2	206.4±53.9	177.3±48.6	196.1±81.9	186.8±26.6	127.5±12.5	161.7±42.0
20:5	20.2±6.6	36.6±8.8	31.5±7.5	37.3±15.3	25.6±4.9	15.9±1.0	19.5±5.3
22:6	44.2±15.9	54.6±14.3	49.2±12.2	55.8±21.1	48.2±4.4	33.3±3.6	45.8±14.0

Table S2. Sequence of primers for quantitative real-time PCR.

Target	Primer sequence (5'-3')	Accession number
GAPDH	F: ACCCAGAAGACTGTGGATGG<br>R: CAGTGAGCTTCCCGTTCAG	NM_002046.7
ATGL	F: AGACAAACTGCCACTCTATGAG<br>R: GAACTGGATGCTGGTGTTG	NM_020376.4
ACC	F: GGAACATCCCTACGCTAAACAG<br>R: CTGACAAGGTGGAGTGAATGAG	NM_198838.2
FAS	F: ACAGGGACAACCTGGAGTTCT<br>R: CTGTGGTCCCACTTGATGAGT	NM_004104.5
SCD-1	F: GGGAGTGTGTCTGCTGAGTAAG<br>R: GCAAGGACTGTTAGAAATCCG	NM_005063.5
